# Targeting the Hallmarks of Aging with Vitamin D: Starting to Decode the Myth

**DOI:** 10.3390/nu16060906

**Published:** 2024-03-21

**Authors:** Carmelinda Ruggiero, Laura Tafaro, Luisella Cianferotti, Flavia Tramontana, Ilaria Giovanna Macchione, Carla Caffarelli, Agostino Virdis, Marika Ferracci, Giuseppe Rinonapoli, Patrizia Mecocci, Nicola Napoli, Valeria Calsolaro

**Affiliations:** 1 Geriatric and Orthogeriatric Units, Division Gerontology and Geriatrics, Department of Medicine and Surgery, University of Perugia, 06156 Perugia, Italy; ilaria.macchione@ospedale.perugia.it (I.G.M.); marika.ferracci@ospedale.perugia.it (M.F.); patrizia.mecocci@unipg.it (P.M.); 2 Division of Internal Medicine, Department of Molecular Medicine, Sapienza University of Rome, 00185 Rome, Italy; laura.tafaro@uniroma1.it; 3 Bone Metabolic Diseases Unit, Department of Experimental, Clinical and Biomedical Sciences “Mario Serio”, University of Florence, University Hospital Careggi, 50134 Florence, Italy; luisella.cianferotti@unifi.it; 4 Unit of Endocrinology and Diabetes, Department of Medicine, Campus Bio-Medico University of Rome, 00128 Rome, Italy; f.tramontana@unicampus.it; 5 Division of Internal Medicine, Department of Medicine, Surgery and Neuroscience, University of Siena, 53100 Siena, Italy; carla.caffarelli@unisi.it; 6 Geriatrics Unit, Department of Clinical & Experimental Medicine, University Hospital of Pisa, 56124 Pisa, Italy; agostino.virdis@unipi.it (A.V.); valina82@gmail.com (V.C.); 7 Orthopaedics and Traumatology Department, University of Perugia, 06129 Perugia, Italy; giuseppe.rinonapoli@unipg.it; 8 Division of Clinical Geriatrics, Department of Neurobiology, Care Sciences and Society, Karolinska Institutet, 17177 Stockholm, Sweden; 9 Unit of Endocrinology and Diabetes, Department of Medicine, Fundation Campus Bio-Medico University, 00128 Rome, Italy; n.napoli@unicampus.it

**Keywords:** aging, vitamin D, hallmarks of aging, clinical studies

## Abstract

Aging is the result of several complex and multifactorial processes, where several agents contribute to an increased intrinsic vulnerability and susceptibility to age-related diseases. The hallmarks of aging are a set of biological mechanisms that are finely regulated and strictly interconnected, initiating or contributing to biological changes and anticipating several age-related diseases. The complex network of cellular and intercellular connections between the hallmarks might represent a possible target for the research of agents with pleiotropic effects. Vitamin D (VitD) is known to have a positive impact not only on muscle and bone health but also on several extra-skeletal districts, due to the widespread presence of Vitamin D Receptors (VDRs). VitD and VDR could be molecules potentially targeting the hallmarks of the aging network. To date, evidence about the potential effects of VitD on the hallmarks of aging is scarce in humans and mainly based on preclinical models. Although underpowered and heterogeneous, in-human studies seem to confirm the modulatory effect of VitD on some hallmarks of aging and diseases. However, more investigations are needed to clarify the pleiotropic effects of VitD and its impact on the hallmark of aging, hopefully highlighting the courses for translational applications and potential clinical conclusions.

## 1. Introduction

Aging is a complex and multifactorial process, marked by a progressive decline in organ functions and an increased risk of age-related diseases and mortality [1,2]. At the biological level, it is a universal process characterized by accumulating damages linked to different mechanisms and pathways defined as “hallmarks” of aging [3]. These hallmarks manifest not merely during physiological aging; they exhibit tight connections with age-related changes, often anticipating alterations associated with age-related diseases and clinical phenotypes years in advance. Therefore, a better understanding of the features and mechanisms related to the hallmarks of aging becomes crucial for the early identification, prediction, and modulation of trajectories of organs and systems’ age-related structural and functional changes. Moreover, experimental manipulation of hallmarks aligns with exacerbating or reducing aging processes and disease-related pathological changes.

To date, mammalians are characterized by twelve biological hallmarks of aging, namely genomic instability, telomere attrition, epigenetic alterations, loss of proteostasis, disabled macroautophagy, deregulated nutrient sensing, mitochondrial dysfunction, cellular senescence, stem cell exhaustion, altered intercellular communication, chronic inflammation, and dysbiosis [2]. These are biomolecular, time-dependent manifestations of alterations and mechanisms accompanying the aging process, enabling aging acceleration experiments and possibly deceleration interventions [4].

While these hallmarks have been extensively discussed in the following section, it is essential to recognize that they do not act as a single independent cause. They are incredibly complex and highly interrelated, complementing others in explaining some or all features of the aging and age-related disease processes [5]. Individually or synergistically, hallmarks can lead to molecular and cellular damage due to primary, antagonistic, and integrative mechanisms. The cumulative effects of the damages are universally adverse and might contribute to the initiation and progression of age-related diseases.

Understanding the mechanistic relationship among the hallmarks and identifying interventions that modulate such processes hold promise for developing strategies to intervene in human aging at the earlier onset of such changes and thus prevent age-related diseases.

Vitamin D (VitD) is renowned for its positive impact on musculoskeletal structure and functions, particularly in preventing falls, fragility fractures, impaired locomotion, and mobility disability among older adults with deficient plasma levels [6]. In the aging process, the decline in physical performance is recognized as the first phenotypical clinical feature of accelerated aging, preceding and contributing to the onset of muscle-skeletal chronic diseases and the burden of disability [7,8,9]. Consistently, aging is associated with a higher likelihood of vitamin D deficiency and dysregulated vitamin D function due to reduced sensitivity to 1,25(OH)2D3. This results from the combined effect of age-related decline in (Vitamin D Receptor) VDR expression, impairment of cutaneous vitamin D synthesis, and altered expression of vitamin D metabolic enzymes. In addition, poor nutrition and reduced exposure to sunlight are commonly seen in long-term care recipients—a population at a particularly high risk of deficiency [10]. Therefore, the clinical practice guidelines state that serum VitD levels of less than 25 nmol/L should be considered as vitamin D deficiency (hypovitaminosis D), 25–50 nmol/L as insufficiency, and over 50 nmol/L as sufficient [11]. Beyond its skeletal benefits, extensive preclinical research and some clinical studies support the extra-skeletal advantages of VitD at multi-organ levels through the 1α-hydroxylase enzyme, which is present in many tissues besides bone, including endothelial cells, vascular smooth muscle cells, cardiomyocytes, pericytes, neural stem cells, neurons, astrocytes, microglia, fibroblasts, osteoblasts, epithelial cells adipocytes, myocytes, monocytes, and macrophages.

The endocrine, intracrine, and paracrine actions of VitD may suggest its involvement in attenuating the aging process and the age-related pathological changes associated with multiorgan diseases [12]. Some studies suggest a role for VitD in influencing biological age at the epigenetic level since individuals with low VitD levels are biologically older than people with adequate levels [13]. Furthermore, VitD has been extensively investigated concerning age-related diseases, particularly cardiovascular disorders. Evidence links low levels of VitD to an increased risk of coronary artery disease, myocardial infarction, and heart failure. VitD supplementation has shown protective effects against the development and progression of cardiovascular diseases [14]. Therefore, randomized clinical trials were conducted to test VitD supplementation for preventing or improving non-skeletal diseases related to poor VitD status. Although the results were mixed, they support the multitargeting actions of low levels of VitD, which significantly affect the aging process by regulating cell homeostasis, counteracting oxidative and inflammatory damage, and cellular senescence.

This narrative review aims to summarize the available evidence regarding the effects of VitD on modulating age-related changes, either leading to or preventing diseases and frailty. Ultimately, we seek to determine whether VitD may be a feasible intervention for attenuating the hallmarks of aging, ultimately suggesting a role in promoting healthy aging or decelerating age-related changes anticipating multi-organ diseases.

## 2. Methods

To serve the purpose of this manuscript, we conducted Pubmed and Embase research, using keywords comprehending “VitD” and each hallmark of aging as previously described, including “genomic instability”, “telomere attrition”, “epigenetic alterations”, “loss of proteostasis”, “disabled macroautophagy”, “deregulated nutrient sensing”, “mitochondrial dysfunction”, “cellular senescence”, “stem cell exhaustion”, “altered intercellular communication”, “chronic inflammation”, and “dysbiosis”, by restricting the search to interventions conducted in the human species. We included English literature for the last ten years (the last search was performed in January 2024), with some exceptions for literature about general concepts.

## 3. The Hallmarks of Aging

**Figure 1:**
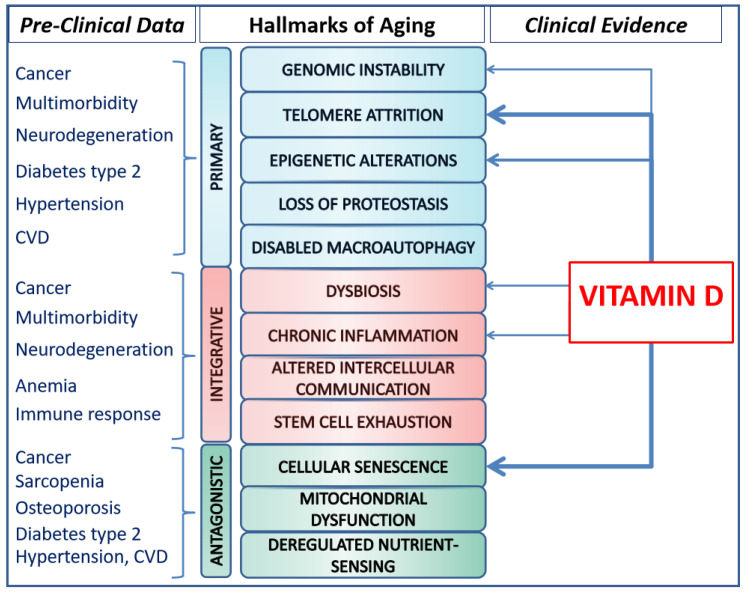
Pre-clinical data supporting the relationship between diseases and hallmarks of aging on the left, and available clinical evidence about the potential impact of Vitamin D on the hallmarks of aging, on the right. Legend: the thickness of the arrows is representative of the amount of available evidence.

Ten years after the initial report, in 2023, Lopez-Otìn et al. revisited the hallmarks of aging, expanding the list of common denominators of aging [2]. The hallmarks, identified across various organisms with special emphasis on mammalians, meet three main criteria. Firstly, they are time-dependent molecular or cellular manifestations of changes or alterations accompanying aging. Secondly, their experimental modulation may accelerate aging processes, and the therapeutic interventions targeting the hallmarks have the potential to decelerate, halt, or reverse age-related processes. The hallmarks of aging are currently categorized as primary, antagonistic, and integrative. Primary hallmarks are the causal agents of cellular damage; they encompass genomic instability, telomere attrition, epigenetic alterations, and loss of proteostasis. Antagonistic hallmarks include deregulated nutrient sensing, mitochondrial dysfunction, and cellular senescence. These initially counteract damages caused by primary hallmarks; however, incidentally or with prolonged pathway stimulation, the antagonistic mechanisms become harmful, contributing to accumulated deleterious age-related changes and processes. Integrative hallmarks, such as stem cell exhaustion, altered intercellular communication, chronic inflammation, and dysbiosis, are interconnected with other damage mechanisms and collectively contribute to functional age-related decline (Figure 1).

The hallmarks of aging are strongly related to each other, exhibiting intricate interactions, with each contributing to and exacerbating the effects of others. Genomic instability crosstalks to epigenetic alterations (e.g., through the loss-of-function mutation of epigenetic modifiers such as TET2), loss of proteostasis (e.g., due to the production of mutated, misfolded proteins), disabled macroautophagy (e.g., through the capacity of autophagy to remove supernumerary centrosomes, extranuclear chromatin, and cytosolic DNA), deregulated nutrient-sensing (e.g., because SIRT6 is an NAD+ sensor involved in DNA repair but also responding to nutrient scarcity), mitochondrial dysfunction (e.g., due to the mutation of mtDNA), cellular senescence (e.g., because DNA damage triggers senescence), altered intercellular communication (e.g., by hampering activation of communication pathways), chronic inflammation (e.g., because CHIP and leakage of DNA into the cytosol induce inflammation), and dysbiosis (e.g., because mutations in intestinal cells favors dysbiosis, whereas specific microbial proteins and metabolites act as mutagens). In addition, senescence can initiate inflammation, which in turn may worsen mitochondrial dysfunction and genomic instability. Epigenetic alterations, on the other hand, can influence gene expression, including the upregulation of proteins like p16 and p21, which promote senescence. Again, the loss of proteostasis, leading to the accumulation of misfolded and damaged proteins, triggers senescence pathways in response to stress. Then, dysregulation of the mTOR pathway, which regulates nutrient sensing, can increase mitochondrial dysfunction and impact on its biogenesis and function. Ultimately, increased ROS release associated with mitochondrial dysfunction can induce epigenetic alterations through DNA methylation and histone modification, which in turn creates a feedback loop of declining mitochondrial function [15]. The interdependence of the hallmarks unveils the complexity of the aging processes and the intricate links between aging and the organ’s thresholds for diseases, and then highlights the need for conceiving aging as a whole and looking for organisms’ macro-biomarkers that are able to quantify the energetic efforts associated with underlying regulatory or compensatory mechanisms leading to diseases [16]. Indeed, whether and how one specific hallmark’s experimental accentuation or attenuation affects other hallmarks does not reveal molecular or cellular beneficial or detrimental effects immediately, given the onset of antagonistic or compensatory mechanisms that, over time, fail, given their intrinsic poor sustainability as disease markers. In addition, evidence is needed to identify whether aging is driven by the deterioration of cellular hallmarks converging on a common aging pathway in all cells or if independent mechanisms will result in different aging pathways in individual cells or tissues. A better understanding of the independence or interdependence of the hallmarks of aging will provide a rational basis for the development of novel therapeutics or interventions to modify the rate of aging.

### 3.1. Genomic Instability

The genomic instability pathway involves accumulating genetic damages caused by exogenous (chemical, physical, and biological) agents and endogenous challenges (DNA replication errors, chromosome segregation defects, reactive oxygen species (ROS), and spontaneous hydrolytic reactions). Such an accumulation of damage causes mosaicism, explaining the coexistence of normal and pathological aging. DNA lesions, such as mutations, deletions, translocations, telomere shortening, single- and double-strand breaks, chromosomal rearrangements, defects in nuclear architecture, and gene disruption caused by the integration of viruses or transposons, impact genes and transcriptional pathways, resulting in dysfunctional cells that compromise tissue and organism homeostasis [2]. When alterations impact stem cells, leading to their exhaustion or hampering their role in tissue renewal, the damage becomes relevant, accelerating the aging process and increasing susceptibility to age-related diseases [17]. DNA repair deficiencies have been linked to aging, suggesting that interventions reducing the mutational nuclear or mitochondrial load and enhancing or rerouting repair mechanisms may slow aging and delay the onset of age-related diseases. VitD emerges as a potential intervention in this context [18].

### 3.2. Telomere Attrition

Telomeres are nucleotide sequences that protect chromosome ends and preserve their genomic stability. Telomeres, together with the sheltering complex, ensure the correct segregation of genetic material during the cell division cycle and prevent DNA repair systems [19]. Due to the inability of the replicative DNA polymerases to complete the copy of telomere regions during cellular division cycles, a shortening of the telomere cap occurs (i.e., telomere attrition) with advancing age, then activating a DNA damage response cascade leading to cellular senescence or apoptosis [20]. Telomerase activity is affected with advancing age, and telomere shortening compromises cell function and life span and is associated with multiple age-related diseases, including cardiovascular disease, malignancies, dementia, osteosarcopenia, frailty, and other conditions [21]. Notably, deficient telomerase activity is also associated with pulmonary fibrosis, aplastic anemia, and dyskeratosis congenita, characterized by a hampered regenerative capacity of the affected tissues. Telomere attrition is adjustable to intrinsic and extrinsic factors, including diet and lifestyle, with evidence for protective effects due to VitD [22].

### 3.3. Epigenetic Alterations

Although the chromosomes carry genetic information, the epigenome is responsible for the functional use and stability of that valuable information, connecting the genotype with the phenotype [23]. Epigenetic alterations represent reversible, heritable mechanisms that occur without altering the underlying DNA sequence. Epigenetic changes can either be spontaneous or driven by external or internal influences, and they may potentially explain different patterns of aging between genetically identical individuals, such as identical twins [24]. A vast array of enzymatic systems is involved in the generation and maintenance of epigenetic patterns, including DNA methyltransferases, histone acetylases, deacetylases, methylases, and demethylases, as well as protein complexes implicated in chromatin remodeling or in ncRNA synthesis and maturation. Then, the major epigenetic alterations affecting aging include alterations in patterns of DNA methylation, abnormal posttranslational modification of histones, aberrant chromatin remodeling, and the deregulated function of non-coding RNAs [2]. Diet interventions, exercise, and pharmacological interventions based on epigenetic-related compounds, including VitD [13,25], are the most accepted strategies to target aging and age-related diseases in humans [14,24].

### 3.4. Loss of Proteostasis

The loss of proteostasis is the mechanism underlying the intracellular or extracellular accumulation of impaired proteins’ aggregates (i.e., misfolded, oxidized, glycated, or ubiquitinylated proteins). Proteome integrity requires tight regulation and crosstalk among distinct proteostasis networks, from translation to degradation. Protein mutations increase their intrinsic tendency to misfolding and aggregation, hence saturating the protein repair, removal, and turnover mechanisms required to maintain a healthy state. In accelerated aging, the burden of misfolded proteins exceeds the capacity of cells to maintain proper proteome integrity and leads to disruptions of cellular function [1]. Over time, the enhanced production of erroneously translated misfolded or incomplete proteins, the slowed translation elongation, and the accumulation of oxidative damaged proteins sustain the collapse of the intracellular network for proteostasis. These alterations increasingly distract the chaperones from folding healthy proteins required for cellular fitness and then increase the failure of the quality control protein response to damage. Several preclinical models found evidence about the loss of proteostasis among neurodegenerative diseases. Dietary VitD supplementation has profound effects on protein homeostasis, with preclinical studies showing profound effects on IRE-1, XBP-1, and SKN-1 functions, suggesting the promotion of protein homeostasis and the slowing of aging processes [26].

### 3.5. Disabled Macroautophagy

Autophagy is an evolutionary, highly conserved, catabolic process that engulfs cytoplasmic materials and dysfunctional organelles by forming a double-layered autophagosome that fuses with the lysosome for the degradation and recycling of nutrients to maintain cellular homeostasis. Autophagy induces stress responses, and starvation, infection, and toxin exposure trigger the catabolic process to protect cells. Alterations in the autophagic functions compromise the intrinsic cellular housekeeping functions, limiting the recycling of metabolic substrates (i.e., free amino acids, carbohydrates, nucleotides, and lipids) for the biosynthetic process for cell maintenance and survival, favoring the accumulation of dysfunctional organelles and protein aggregates, and limiting the clearance of intracellular microbes through lysosomal degradation [27]. Indeed, autophagy primarily serves as a cytoprotective mechanism, and its impairment is associated with neural disorders, developmental abnormalities, inflammatory diseases, aging, and cancer [27]. VitD prompts cellular autophagic activities through genomic and non-genomic signaling, influencing various physiological functions along with calcium metabolism. Autophagic mechanisms regulated by VitD and its receptor exert protective effects in inhibiting oxidative stress and apoptosis, controlling cellular proliferation, differentiation, inflammation, and host immunity by activating antimicrobial defense mechanisms [27].

### 3.6. Deregulated Nutrient-Sensing

The nutrient-sensing pathways detect and respond to fluctuations in environmental nutrient levels. Physiologically, nutrient abundance activates anabolism and storage, whereas scarcity triggers homeostatic mechanisms that mobilize internal stores through autophagy. The nutrient-sensing network includes extracellular ligands (i.e., insulins, IGFs, and receptor tyrosine kinases) with which they interact and intracellular signaling cascades. These cascades involve the PI3K-AKT and Ras-MEK-ERK pathways and transcription factors, including FOXOs and E26 factors, which transactivate genes involved in diverse cellular processes.

Deregulated nutrient-sensing metabolic status is associated with a compromised somatotrophic axis, which includes the growth hormone (GH) and IGF-1, mainly produced by hepatocytes in response to the blood glucose concentration and activating intracellular signaling pathways. In preclinical models, diminished IGF-1 levels and GH/IGF-1 signaling are associated with improved stress defenses, autophagy, and cell survival via reduced PI3K/Akt and mTOR signaling. Moreover, GH receptor deficiency improves the defense from oxidative stress in healthy tissues and promotes apoptosis in neoplastic cells [28]. Modulating or perturbing nutrient-sensing node pathways seems to be a robust way to slow aging processes, extend the human lifespan, and protect against aging-related diseases. Ongoing studies testing caloric restriction (a reduction in caloric intake without apparent signs of malnutrition), pharmaceuticals, dietary patterns, or genetic interventions are waiting for more convincing results [29,30].

### 3.7. Mitochondrial Dysfunction

Mitochondrial structure and dynamics change with aging. Mitochondria from older subjects swell while their numbers decline and cannot replace themselves as quickly as in their optimal functional state [31,32]. Mitochondrial dysfunction follows the accumulation of mitochondrial DNA mutations, the increased production of ROS and related damage to cellular macromolecules, the impaired cellular bioenergetic and higher permeabilization of mitochondrial membranes, causing inflammation, altered stress responses, and cell death (when activators of caspases, nucleases, or other lethal enzymes are released from the intermembrane space). Notably, mitochondrial dynamics and quality control changes promote the accumulation of damaged mitochondria, which contributes to aging and age-related pathologies. In addition, several cardiac and neurodegenerative diseases, muscle atrophy, and sarcopenia recognize alterations in mitochondrial fission and fusion machinery [33]. Strategies aiming to counteract the age-related decline in L-carnitine levels, which may limit fatty acid oxidation by mitochondria, have revealed improvement or rescue of mitochondrial dynamics and quality control defects in a placebo-controlled trial on pre-frail subjects and older men [34]. Mitochondrial-derived peptides are emerging as key players in mitochondrial dysfunction through interactions with stress-sensitive transcription factors. Humanin is a microprotein encoded by mtDNA that declines with age, exhibits high levels in centenarians and their offspring, and emerges as a potential anti-aging factor linking organellar function to organismal homeostasis [35]. Humanin lacks contributions to cellular senescence, chronic inflammation, and cognitive decline [36]. Concerning mitochondrial dysfunction, VitD directly promotes and prevents protein oxidation, lipid peroxidation, and DNA damage and indirectly regulates autophagy, inflammation, epigenetic modifications, DNA abnormalities, and calcium and ROS signaling changes [37].

### 3.8. Cellular Senescence

Senescence is a state of permanent cell cycle arrest in response to different damaging stimula. During development, adulthood, and after injury, senescent pathways play a pivotal role in tissue remodeling, acting as a positive regulator of the regenerative potential and function of tissues. Senescent cells are also a strong safeguard against tumorigenesis, pacing irreversible proliferation arrest via cell and non-cell autonomous pathways. In aged organisms, the accumulation of cellular senescence and the release of secreted factors (SASP) adversely modify the behavior of senescent and non-senescent cells, remodel the extracellular environment, and sustain inflammation and tumorigenesis. Dysfunctional senescent cells may be powerful targets for slow-aging approaches, but the existence of beneficial senescence programs complicates the development of interventions without incurring toxicities [38]. Nutrition and a healthy lifestyle are intimately linked and can greatly impact cellular senescence. Nutritional elements can influence cellular senescence, with evidence for VitD from both in vitro and in vivo research [39]. In preclinical models, interventions targeting senescent cells have been shown to delay, prevent, or alleviate tissue damage and multiple disorders. Senolytic drugs may target selectively senescent cells, inducing their lysis, leading to promising strategies for preventing or treating multiple diseases and age-related conditions in humans. However, in some cases, their molecular targets have not been precisely identified and characterized, making them hard to introduce in clinical practice in the short term [40].

### 3.9. Stem Cell Exhaustion

Stem cell exhaustion refers to the numerical decline and impaired dynamics of the constitutional stem cells; both aspects are crucial strategies for living organisms. With unstable populations of proliferating stem cells, tissues and organs lose their ability to recover from primary or injury-induced damage and begin to fail. Cellular de-differentiation, plasticity, and cellular reprogramming, i.e., the plasticity of resident stem cells under normal conditions, are the main mechanisms for tissue renewal and repair. While stem and progenitor cells are all subject to the same hallmarks of aging, the mechanism of cellular reprogramming is thought to act autonomously on multiple cell types, with higher relevance for the long-term impact on tissue rejuvenation. Cellular re-programming consists of the conversion of adult somatic cells into embryonic pluripotent cells; transient reprogramming confers repair capacity to old tissues so that subsequent damage is repaired as efficiently as in young individuals; partial reprogramming encompasses features of the natural process of tissue repair, according to the finding that the epigenetic methylation clock accelerates soon after tissue injury and partially reverses during tissue repair [2]. A large body of the preclinical literature reports stem cell-based interventions’ therapeutic impacts on tumors, fibrotic disorders, and tissue damage [41]. Human trials of any stem cell therapy must be approached cautiously due to the risk of genetic and epigenetic alterations occurring during their induction and manipulation. So far, insufficient clinical evidence supports the effectiveness and safety of anti-aging stem cell infusions [42]. Therefore, we are waiting for findings from trials looking for the effects of stem cell-based interventions on physical frailty and skin aging, which are still in their development phase [43].

### 3.10. Altered Intercellular Communication

Aging is characterized by altered intercellular communication due to extensive changes in the amount and secretion of cellular factors and hormonal signaling pathways. On one side, neural, neuroendocrine, and hormonal signaling pathways decline with aging due to intrinsic adaptations, including adrenergic, dopaminergic, and insulin/IGF1-based and renin-angiotensin systems and sex hormones. On the other side, intrinsic cellular alterations and environmental cellular exposures increase the SASP (senescent-associated secretory phenotype) and extracellular vesicles (EVs), acting as carriers for intercellular messaging through miRNAs that progressively impact the function of neighboring and distant cells, modifying the body’s coordinated functions and responses. The intercellular communication derangements ultimately sum up the hallmarks of aging on its own bridging the cell-intrinsic hallmarks to meta-cellular hallmarks, including chronic inflammatory reactions, impaired immunosurveillance against pathogens and premalignant cells, and alterations in the bidirectional communication between the human genome and microbiome. The main focus in this area is exploring the effects of blood-borne systemic factors on pro-aging or pro-longevity properties. VitD is a fine-tuned immunomodulatory molecule that impacts immunosenescence and inflammaging, with evidence showing that adequate VitD levels help counteract the natural decline in immune surveillance [44].

### 3.11. Chronic Inflammation

Inflammation is a ubiquitous mechanism evolved to protect an organism from infection and injury. While the inflammatory cascade in response to acute infection or injury clears invading pathogens and incites wound healing, chronic inflammation is a potentially pathologic process arising from the perpetuity of the initial trigger or the dysregulation of signaling pathways that is harmful to health. The term “inflammaging” has been coined to describe such a low-grade, chronic inflammation process that progresses with age and impacts several processes. Chronic inflammation occurs because of multiple derangements stemming from the hallmarks of aging, and then it progressively increases according to different trajectories depending on the spatial–temporal degree of interaction between coexistent hallmarks and intrinsic and extrinsic factors [45]. The VitD immunomodulatory role is widely recognized to have positive effects on health span and lifespan. An adequate level of VitD counteracts inflammation with multilevel targeting effects, i.e., inhibiting the expression and signaling of TLR2, 4, and 9, reducing the production of cytokines such as TNF-a, IL-6, and Il-23, and repressing the activity of T cells recruiting chemokines [46]. The combination of VitD and curcumin supplements has been recently hypothesized to counteract neurodegeneration [44].

### 3.12. Dysbiosis

Dysbiosis refers to an imbalance in the gut microbiome, which can occur because of several factors related to host genetic variants (ethnicity), dietary factors, and lifestyle habits (culture), as well as environmental conditions (geography), which makes it challenging to unveil the relationships between the microbiota and pleiotropic age-associated disease manifestations. In the context of the human microbiome interaction, dysbiosis negatively impacts health, acting as a catalyst factor for fueling inflammation. Moreover, interventions aimed at restoring a youthful microbiome may extend health span and lifespan, with several inflammatory microbial metabolites, including SCFAs, and exopolysaccharides microbially derived, downregulating gut inflammation [47]. Caloric restriction diets induce structural changes in the gut microbiome, increasing the abundance of Lactobacillus and other species that influence healthy aging. A randomized, double-blind, placebo-controlled pilot study in overweight/obese insulin-resistant volunteers showed that the oral administration of pasteurized A. muciniphila improved insulin sensitivity and reduced insulinemia and plasma total cholesterol levels [48]. Notably, the composition of the intestinal microbiome could be altered by vitD deficiency, and supplementation has been determined to improve gut microbiomes with a positive impact on their overall health and immune system function. Ultimately, fecal microbiota transplantation opens the possibility of manipulating the gut microbiota with pre-, pro-, and post-biotics to rejuvenate the immune system and connected organs, prospecting the analysis of microbiome data for personalized patient care [49].

## 4. Vitamin D and Its Potential Impact on the Biological Hallmarks of Aging

VitD is a highly regulated fat-soluble hormone, and biosynthesis starts after subcutaneous UV irradiation of 7-dehydrocholesterol. UV radiation plays a crucial role in initiating the conversion of 7-dehydrocholesterol to pre-vitamin D3, which further transforms into cholecalciferol, following the mevalonate pathway and the Kandutsch–Russell branch of the sterol pathways. Interestingly, VitD shares common metabolic pathways with cholesterol; their biosynthesis starts with acetyl coenzyme A (AC-CoA) in the cytosol and recognizes the influence of similar SNPs [50]. Cholecalciferol then converts into calcidiol (25(OH)D) after liver metabolic activation and finally into the active form calcitriol (1,25(OH)2D). The active form of VitD is involved in several complex metabolic pathways, occurring in several cellular types, and spanning extracellular regions, cytosol, lysosome, endoplasmic reticulum, and mitochondria. Both circulating calcidiol and calcitriol bind to the VitD binding proteins (VitD-BP), then calcitriol dissociates in tissues to bind to the VDR, a nuclear receptor transcription factor, and calcidiol seems able to bind accessory pockets of the VDR [51]. The calcitriol uptake into cells occurs in both bound and unbound forms and regulates calcium and phosphorus homeostasis through binding to VDR [52]. Beyond bone metabolism, VitD-BPs have immunomodulatory functions, being a negative acute phase protein with levels dropping by about a third in patients with acute health conditions [53]. Notably, VDR seems essential for mitochondrial bio-energetic function through the regulation of key mitochondrial gene sets. The role of single nucleotide polymorphisms (SNPs) in the VDR gene has been largely investigated but yet makes definitive conclusions difficult due to variable methodologies and different demographics among study populations [54]. Additionally, no evidence exists proposing a molecular basis for the differences seen between those who carry VDR SNPs and those who do not; clarification of the mechanisms underlying the functional consequences of these SNPs would provide further information into the role of the vitamin D/VDR axis and the extent to which its genetic component contributes to our physiology [55].

Considering the wide range of functions that VitD exerts on many cellular systems, it would not be surprising to find an impact on the hallmark of aging. However, the literature about the potential role of VitD on hallmarks of aging is scarce, limited to a few clinical studies and mainly preclinical models, probably due to the complexity of pathways involved in the aging process. Nevertheless, the fact that VitD exerts genomic actions has been known for a few years now. VitD acts through binding to VDR, after which heterodimers with retinoid receptors are created, and interacts with VitD response elements (VDREs), enhancer elements in DNA. Indeed, classic VDR belongs to the nuclear receptors and directly affects gene expression. It could induce gene transcription and also suppress gene expression, depending on the VDR interactions [56]. In addition, 1,25-dihydroxyVitD may also act through a receptor in the cytoplasmic membrane associated with a special type of lipid rafts called caveolas. Through the VDR in the cytoplasmic membrane, 1,25-dihydroxyVitD can activate signaling pathways and elicit a rapid cellular response [57]. The wide gene targeted by VitD and VDRs justifies the pleiotropic functions of VDRs. Table 1 summarizes the main human studies investigating the effects of VitD supplementation on the main hallmarks of aging. A detailed description of the studies is reported and discussed in the following sections.

### 4.1. Vitamin D and Genomic Instability

Associations between VitD and markers of DNA integrity and stability have been mainly presented in studies conducted in human cultured cells and observational studies conducted in patients with comorbid diseases, such as Type 2 Diabetes Mellitus (T2DM), obesity, infertility, cancer patients, and the general population [2,58,59]. Oncogenic-induced senescence has been the drive to demonstrate VitD and VDR genomic functions. Without going deeply through oncogenic pathways, oncogene-induced senescence is the consequence of the expression of oncogenic RAS, as a tumor suppressor mechanism. In more detail, the expression of RAS leads to the down-regulation of VDR, which in turn determines a reduction in BRCA1 and 53BP1, which are the main actors involved in genome stability. The levels of those factors appear to improve with VitD supplementation [60]. In a placebo-controlled trial involving 92 patients with a history of colorectal adenoma, there was no clear evidence of a reduction in 8-hydroxy-2-deoxyguanosine, a marker of DNA damage in normal colorectal epithelial crypt cells. The daily administration of 800 IU VitD3 alone for six months led to a significant increase in Bax expression, an apoptosis promoter, without changes in Bcl-2 expression, an apoptosis inhibitor [61]. However, a randomized control trial conducted in 92 people with VitD insufficiency (<30 ng/mL) showed that VitD supplementation (2000 IU daily for 3 months) decreased the percentage of DNA damage and oxidative parameters when compared to the control group, either with or without diabetes [62].

A potential role of VitD in modulating genomic instability has been assessed in T2DM. Indeed, poor glucometabolic control might affect genomic stability; at the same time, VitD plays a role in sustaining DNA damage repair. In their study, Fagundes et al. aimed to evaluate the effect of VitD in improving glucose and lipid metabolism as well as the modulation of the genomic instability derived from T2DM. The Damage Index obtained from a comet essay was reduced during VitD supplementation therapy, together with an anti-mutagenic action. VitD supplementation led to a significant decrease in nitric oxide (NO) and total thiols and an increase in the concentration of reduced glutathione (GSH), leading to a decrease in oxidative processes in cells. Even though this result might be debated, it is important that the antioxidant function of VitD may be a protective mechanism against DNA damage [63].

### 4.2. Vitamin D and Telomere Attrition

Several human studies have aimed to elucidate the relationship between VitD and telomere biology. A positive correlation between serum 25(OH)D levels and telomere length was found in peripheral blood leukocytes among women in the twins UK cohort. Even after adjusting for various factors, the difference in telomere length between the highest and the lowest 25(OH)D tertiles equated to a 5-year difference in telomeric aging [64]. The Nurses’ Health Study, conducted on 1424 women, confirmed a positive correlation between telomere length and serum 25(OH)D concentrations. However, no significant association was found with 1,25(OH)2D3 levels. Genetic factors related to VitD metabolism did not significantly affect telomere length [65]. However, the association between 25(OH)D or 1,25(OH)2D3 levels and peripheral blood leukocyte telomere length was not confirmed in 2483 men [66] and 5096 young adults from the Northen Finland Birth Cohort 1966 [67]. Nevertheless, the study identified an inverse association between body mass index (BMI) and telomere length, even after adjusting for various factors, including 25(OH)D levels [67].

A more recent analysis conducted in older adults (≥85 years old) examined the association between 25(OH)D concentration and telomere length in peripheral blood mononuclear cells (PBMCs) over time. They found a significant positive association at baseline but inconsistent relationships at subsequent time points [68]. Focusing on patients with systemic lupus erythematosus and healthy controls with VitD deficiency, again a significant correlation appeared between telomere length and serum 25(OH)D concentrations [69]. Ultimately, active VitD treatment was associated with a greater telomere length in PBMCs in a small sample of hemodialysis patients, supporting its potential protective role [70]. The effect of an intervention based on the combination of regular-strength exercise and supplements that included branched-chain amino acids (BCAAs), calcium, and VitD was tested on the telomere length and the expression of telomeric repeat-containing RNA (TERRA). Such an intervention significantly increased TERRA but did not alter telomere shortening [71]. TERRA is central to the modulation of telomere length. In cells with longer telomeres, TERRA competes with the telomerase’s DNA substrate or it enhances the catalytic reverse transcriptase subunit of the enzyme to inhibit the elongation of telomeric repeats [72]. On the contrary, in cells with shorter telomeres, TERRA promotes telomere lengthening by facilitating the recruitment of telomerase. It is possible that the findings reflect a lag in the increase in telomere size. The cross-sectional design of this study limits any causal associations between TERRA and telomere length, which need to be explored using a prospective study design. By using summary-level data from the largest genome-wide association studies, a Mendelian randomized study investigated the causal relationship between serum VitD (*n* = 73,699) and telomere length (*n* = 37,684). The findings did not support any causal effect of VitD on telomeric length [73].

Obesity is a condition characterized by low-grade chronic inflammation with increased oxidative stress status, which in turn negatively impacts telomerase wellness. It has already been demonstrated that obesity accelerates aging and is associated with shorter telomeres. Telomerase activity maintains telomere lengths and prevents T cell senescence, preserving long-term immune function. On this basis, Zhu et al. recently evaluated whether VitD supplementation might increase telomerase activity in a population of overweight African American subjects compared to the control group. Telomerase activity was measured using the Telomeric Repeat Amplification Protocol (TRAP). At baseline, PBMC telomerase activity did not differ between the two groups, but VitD supplementation significantly increased telomerase activity, persisting after an adjustment for sex, BMI, and age, supporting the idea of a beneficial effect of VitD on telomer wellness [74]. However, telomere length was not measured in this study [74].

### 4.3. Vitamin D and Epigenetic Alterations

On a different note, the concept of epigenetic alterations leading to accelerated aging has been developed in recent years and has been associated with several diseases, such as obesity, Werner syndrome, Huntington’s disease, and Down syndrome. The cumulative effects of epigenetic alterations are measured as the DNAm (DNA metilation) and seem to be highly correlated with the individual’s chronological age [75]. The possible role of VitD in modulating epigenetic regulation has been hypothesized, considering the association between global DNA hypometilation, leucocyte DNA metilation changes, and increased global metilation levels after VitD administration [76,77]. In a recent study conducted to evaluate the effect of VitD on epigenetic aging in a cohort of young, obese African women, DNAm age was correlated with chronological age in patients with low VitD levels, while the supplementation decreased DNA metilation and slowed epigenetic aging [75]. Pregnancy has been another condition of interest for the study of epigenetics and DNA methylation. As part of the Southampton Women’s Survey (SWS) mother–offspring study, it has been demonstrated that methylation at the retinoid-X-receptor-alpha (RXRA) locus in the umbilical cord was associated with the offspring bone mass. The relevance of this discovery is due to the fact that RXRA is part of VitD intracellular signaling, and greater VitD levels were associated with a lower RXRA methylation burden [78]. From the cohort of the Maternal VitD Osteoporosis Study (MAVIDOS), Curtis et al. measured the DNAm at a specific site in the RXRA locus (10 CpG) and found that in the cohort of mothers receiving cholecalciferol, the methylation levels in the RXRA-specific site were significantly lower compared to the placebo group [79].

Anderson et al. implemented an epigenome-wide approach to identify differentially methylated loci among infants born to mothers supplemented with 400 IU (i.e., control) versus 3800 IU (i.e., intervention) daily of VitD. Among mothers, supplementation with 3800 IU of VitD per day was associated with mean 25(OH)D levels reflective of a sufficient maternal VitD status by birth among the intervention group, as well as significant gains in maternal leukocyte DNA methylation associated with genes involved in cell migration/motility, development, and growth. The implications of methylation gain for gene expression and pathway activity could be related to blood vessel development, which is central to the biological processes associated with the demands for a hypertrophic maternal circulatory system. The continued development of the circulatory needs of the placenta and maternal VitD status have been shown to affect placental vascular endothelial growth factor gene expression. Among infants, those born to mothers in the intervention group showed methylation gain at loci with biological implications for the developmental processes, including bone, lung, metabolic, and nervous systems. Among infants of mothers in the intervention group, methylation loss was associated with genes known to play a role in metabolic processes and signal transduction pathways, a finding also noted in examining the leukocytes of their mothers [80].

### 4.4. Vitamin D and Lack of Proteostasis

How VitD participates in maintaining proteostasis has been studied in the preclinical model of C. Elegans, in which DAF-16, HSF-1, and SKN-1 are associated with the premature accumulation of insoluble proteins and alterations of protein homeostasis. In their study, Mark et al. found that VitD feeding was able to suppress the toxicity of amyloid-beta and slow protein insolubility, reducing protein toxicity in favor of longevity [26]. Preclinical studies have shown a role of VitD in muscle proteostasis, with a fine regulation in place between muscle protein synthesis (MPS) and catabolism (muscle protein breakdown: MPB). The ubiquitin-proteasomal pathway (UPP) is one of the proteolytic systems where VitD plays a recognized role. In fact, the deficiency of VitD in rats leads to increased MPB and increased expression of ubiquitin conjugating enzyme and ubiquitin conjugates, with no increase in lysosomal enzymes; as a result, VitD deficiency seems to contribute to increased non-lysosomal proteolysis [81]. Moreover, the lack of VitD signaling leads to muscle atrophy of type II fibers [82], sustaining the critical role of VDR in muscle metabolic flexibility and pancreatic insulin response. Recent studies have suggested that VDR-deficient mice are predisposed to utilize fatty acids as the primary energy source and accordingly exhibit an increase in the oxidative type I and type IIa fibers and atrophy affecting both of these muscle fiber types [83]. On the other hand, VitD has a role in the phosphorylation of mitogen-activated protein kinase (MAPK) pathway, influencing cellular signaling involved in myogenesis [81]. Adenosine monophosphate-activated protein kinase (AMPK) represents another proteostasis regulator that is linked to VitD levels. Indeed, AMPK is activated during metabolic stress, acting as a nutrient sensor and positively regulating the cellular response to stressors. It is known that AMPK expression declines with age, but in vitro studies suggest that its activity seems to increase after treatment with 1,25(OH)2D3. Moreover, VitD-deficient rats had reduced activation of sirtuin-1, whose activity was derived from AMPK activation [55]. So far, evidence in this regard comes from preclinical models, but considering the widely recognized role of VitD supplementation for muscle health, some analogue mechanisms in humans could be hypothesized.

### 4.5. Vitamin D and Mitochondrial Dysfunction

Protein homeostasis and cellular respiration should not be considered separately. The finding of novel mitochondrial localization of VDR has shed light on the impact of VitD on mitochondrial function. Indeed, after binding VRD, VitD seems able, in preclinical models, to reduce mitochondrial respiration, consequently preventing the excess of ROS [84]. In aged mice, the expression of the protein Nrf2 (nuclear factor erythroid 2–related factor 2, a regulator of cellular defense from oxidants), which is known to improve mitochondrial function, is reduced, but increases after treatment with calcitriol, further confirming the role of VitD in mitochondrial homeostasis [85]. The regulation of mitochondrial function has been seen as helpful in reversing age-related hypertension in young male mice [85]. Again, due to the known role of mitochondria in neurodegeneration and Alzheimer’s disease in particular, Mohanad et al. found that VitD was able to improve the oxidative stress and mitochondrial function in a preclinical model of induced AD, through calcium/calmodulin-dependent protein kinase kinase 2 (CAMKK2)-mediated phosphorylation of Sirtuin1 (SIRT1) [86].

One of the districts that highly depends on adequate energy production and cellular respiration is the muscular one. In chronic obstructive pulmonary disease (COPD), besides the pulmonary impairment, several extra pulmonary districts are involved. In particular, type I muscle fibers suffer from reduced airflow and oxygenation, and the muscular structure switches from slow to a fast-glycolytic metabolism. Thus, increased fatigue and reduced endurance lower extremity strength with consequent impaired mobility, and finally, sarcopenia or cachexia represent possible consequences [87]. VitD deficiency has been demonstrated to be linked to mitochondrial dysfunction, ATP depletion, increased production of ROS, with the known damage that oxidative stress causes to muscular tissue, and VitD supplementation, which has a positive impact on improving lung function, inspiratory muscle strength, and oxygen uptake. Ca^2+^ homeostasis is a key player in muscle health; moreover, Ca^2+^ represents, together with ROS, secondary messages in cellular signaling. VitD is the main regulator of Ca metabolism, and its deficiency may impair calcium uptake in mitochondria, compromising adequate homeostasis. Again, VitD plays a role in protein synthesis degradation and peroxidation, actively impacting the ATP-ubiquitin-dependent system and nitrosative stress [87]. Several other examples of evidence support the VitD beneficial effect on the attenuation of mitochondrial oxidative stress. In rats, VitD attenuated the oxidative stress neurotoxicity induced by cyanide. In rats’ cardiovascular systems, VitD was proven efficient in reversing the oxidative cardiac injury induced by isoproterenol, reducing H_2_O_2_ levels, and increasing anti-oxidant mechanisms. Moreover, VitD modulates the expression of monoamine oxidase, known to be a mitochondrial pro-oxidative enzyme, and restores vascular function [88]. The VitD beneficial function is exerted through VDRs activation; in mouse hearts, VDR’s activation was able to reduce tissue ischemia/reperfusion stress, and its overexpression ameliorated the myocardial infarct size and increased cardiac function.

Improvement in mitochondrial stress with VitD has also been seen in the central nervous system; in fact, pre-treatment with calcitriol showed attenuation of oxidative stress and ROS production in conditions of mild hyperomocysteinemia. The same protective effect was demonstrated in the heart tissue when exposed to the same condition. Interestingly, VitD combined with lipoic acid reduced mitochondrial stress in astrocytes and neurons, both activated by H_2_O_2_ and streptozotocin [88]. With aging, the accumulation of AGE leads to several alterations in the cardiovascular system, such as stiffness, atherosclerosis, plaque formation, and endothelial dysfunction. In cardiomyocyte cells treated with AGE, mitochondrial dysfunction was associated with contractile impairment. Treatment with VitD reduced AGE accumulation in rats, therefore suggesting a further potential protective role [89]. Mitochondrial dysfunction, and consequently, altered placental metabolism and increased ROS production, is a feature widely recognized in obese pregnant women; similarly, obese women seem to be more frequently VitD-deficient. Phillips et al. evaluated whether trophoblast cells treated with VitD could show an improvement in mitochondrial respiration: in vitro treatment with calcitriol showed an increased expression of VDR, both in obese and non-obese women, while no significant difference was found in oxygen respiration after treatment in non-obese women, and some improvement was seen in obese ones [90]. Crohn’s disease (CD) represents a chronic invalidating disease, and intestinal fibrosis is a common complication. Epithelial cells switch to mesenchymal cells through a process called epithelial–mesenchymal transition (EMT). Aside from the knowledge that patients with CD have reduced VitD levels and that VDD could lead to intestinal fibrosis, Yu et al. investigated whether VDR might have a role in modulating intestinal fibrosis through mitochondrial regulation. In VDR-knockout mice, epithelial mitochondria showed altered morphology, accumulation of ECM in the interstices, and reduced functional genes, suggesting a potentially protective role of VDR activation against intestinal fibrosis [91]. Finally, sarcopenia represents a widely recognized clinical syndrome, characterized by impaired physical performance and related to poor outcomes. The role of VitD in sarcopenia has been evaluated since hypoVitD is linked to muscle weakness, reduced strength and mass, fatigue, and reduced mitochondrial activity. However, the results from intervention studies evaluating the role of VitD are conflicting and inconclusive, recommending further studies to better understand and define the possible role of VitD supplementation in sarcopenic patients [92].

### 4.6. Vitamin D and Cellular Senescence

There are several insults that might activate the cellular senescence process. When the cell reduces proliferative capacity, it remains in the Eg1-g2 PHASE, and then interrupts divisions and changes morphology. The hallmark of senescence is the senescence-associated secretory phenotype (SASP). Proinflammatory cytokines released by the secretome may increase and trigger secondary senescence in the surrounding environment. Again, VitD supplementation seems to be related to decreased cellular senescence and SASP [93]. Part of the aging process is related to the aging of the immune system due to thymic involution and function and the increased number of T immature T lymphocytes, with the immune phenotype changing towards a lower CD4/CD8 ratio and a decreasing amount of T CD8+CD28+ cells. The inevitable process of immunosenescence exposes older people to recurrent infections and cancers; moreover, chronic disease and low-grade inflammation represent a further step down in immune system efficiency [94].

A recent trial aimed to test the possible effect of a nutraceutical supplement (comprehensive of Sambucus nigra, zinc, tyndallized Lactobacillus acidophilus (HA122), arabinogalactans, VitD, vitamin E, vitamin C, and group B vitamins) on some specific signatures of immunosenescence in hospitalized older adults. These supplements determined a reduction in inflammatory mediators such as IL-6, CRP, and lymphocyte number, and they were also associated with perceived better wellness [55,95]. Considering the effect of VitD on the immune system, mainly exerted through the inhibition of monocyte and cytokine overproduction and direct action on T lymphocytes, Rizka et al. evaluated the potential effect of alphacalcidol supplementation in an inflammatory profile (Il-6, IL-10, TNF, CD4/CD8 ratio, CD8+, and CD28-) in 110 older fit or frail subjects. The population was randomized to alphacalcidol or placebo and administered orally for ninety days. The cohort receiving alphacalcidiol had a better immune profile across the whole cohort as well as the cohort divided according to frailty status [96]. These results support the immunomodulatory function of VitD and highlight the importance of maintaining sufficient levels of VitD, which seem crucial in immunosenescence and chronic conditions characterized by low-grade chronic inflammation. In addition, calcium and calcium signaling have been established as critical factors in the implementation and regulation of cellular senescence. Increased intracellular calcium accumulation occurs in different cell types and in response to many different senescence-inducing stimuli. In senescent cells, intracellular calcium concentration increases in the cytosol and mitochondria, coming from the extracellular space through plasma membrane channels, the release by the endoplasmic reticulum, and mitochondrial dysfunction. Calcium fluxes between different subcellular compartments, calcium channels, calcium-binding proteins, calcium-regulated enzymes, and transcription factors also play pivotal roles in promoting senescence. However, a thorough assessment of the functional contribution and regulation of each of the factors involved in calcium signaling is still needed to fully understand it [97].

### 4.7. Vitamin D and Stem Cell Exhaustion

The influence of VitD on stem cells (SCs) mainly refers to in vitro studies conducted on fetal or adult osteoblasts. The administration of VitD per se or loaded onto nanoparticles and other nanostructures is associated with great potential in bone regeneration and improved stem cells’ differentiation into osteoblasts [98].

VDR signaling also plays a vital role in the modulation of neuronal SCs, which exhibit a prominent role in developing the entire nervous system during the embryonic stage; even in adult brains, neuronal SCs are located in restricted regions and support the replacement of damaged neurons [99]. VDR-mediated cellular signaling cascades are involved in enhancing neuronal SCs through Wnt/β-catenin and Sonic Hedgehog pathways. Based on the canonical signaling of Wnt in activating neurogenesis in the brain and maintaining balance between activated neural stem cells (aNSC)/qNSC avoiding depletion, it is crucial to maintain persistent VDR activity. Therefore, deficiency in VitD could cause an imbalance in Wnt signaling and inhibit the gradual neural SC stimulation required to maintain a neurogenic rate [100]. However, more research is needed in this field to identify potential VDR agonists targeting neuronal SCs. More recently, preclinical studies identified a potential role of VitD on SCs from patients with acute myeloid leukemia (AML). These patients usually display insufficient or deficient VitD levels, which are significantly associated with poorer overall survival, both at diagnosis and before allogeneic stem cell transplantation. There is seminal evidence from animal studies that VDR agonists may impair leukemic stem cell activity, while the lack of VDR results in increased numbers of hematopoietic and leukemia stem cells and quiescent hematopoietic stem cells, suggesting a function of VitD as regulators of stem cell homeostasis [101,102]. However, therapeutic interventions with VitD analogues have resulted in inconclusive data in humans due to adverse events related to systemic hypercalcemia [103]. Possibly more promising results could be derived from recent pilot studies evaluating the effects of adding VitD to the adipose tissue-derived stromal/stem cell (ASC) infusion in patients with Type 1 Diabetes [104].

### 4.8. Vitamin D and Altered Intercellular Communication

Very scarce literature is available regarding the possible role of VitD in intercellular communication, and it is mainly focused on bone cells. It is already known that fibroblasts and other osteoblast-lineage cells lead to the formation of osteoclasts. However, it is not clear whether intercellular interactions have an impact on the expression of genes implied in osteoclastogenesis. In their study, Bloemen et al. evaluated the effect of the cell–cell interaction on the micro-RNA (mRNA) expression of the molecules involved in osteoclast formation in cell cultures. Moreover, they evaluated the formation of tartrate-resistant acid phosphatase (TRACP)-positive cells as well as their ability to resorb bone. Interestingly, their data showed that osteoclastogenesis-related genes were up-regulated in co-cultures compared to mono-cultures and were overpowered by both VitD and dexamethasone [105]. Intercellular communication is also a contributing factor for receptor expression on osteoblast-like cells, which is fundamental for hormone and neurotransmitter binding and signaling. Several years ago, Schirrmacher analyzed the electric coupling in a gap junction of rat osteoblast-like cells after stimulation with VitD, 17β-estradiol (17β-E2), the neurotransmitter vasoactive intestinal peptide (VIP), and the excitatory amino acid glutamate (Glu). They found that the electric coupling response was cell-pair-specific when exposed to the above-mentioned hormones. Specifically, VitD increased the coupling in some cell pairs but not in others [106]. Considering the pleiotropic actions of VitD at cellular levels, it could possibly have an influence on intercellular communication as well, and more studies are warranted to better address this topic.

### 4.9. Vitamin D and Chronic Inflammation

VitD influences the innate and adaptative immune responses given to the wide distribution of VDR among immune cells and their intrinsic ability to convert VitD metabolic precursors into the active form, and then allows for an autonomic regulation of the active VitD concentration in an inflamed site [107]. Although several preclinical and epidemiological studies confirmed the inverse relationship between VitD and chronic inflammation, intervention studies addressing the impact of VitD supplementation on low-grade inflammation are not consistent. Indeed, VitD supplementation reduces the high-sensitivity C-reactive protein (hs-CRP) in patients with diabetes [108], psychiatric disorders [109], polycystic ovary syndrome [110], and advanced kidney disease [111], and reduces the tumor necrosis factor-α (TNF-α) in patients with diabetes [112]. However, a systematic review of meta-analyses and randomized controlled trials (RCTs) found that VitD3 supplementation at a range of doses had no significant effect on the biomarkers of systemic inflammation and hypothesized that a low VitD status is a consequence of ill health rather than its cause [113]. The VITAL study aimed at the primary prevention of cancer and CVDs and was conducted among 25,871 women ≥ 55 and men ≥ 50 years of age, with a mean treatment period of 5.3 years. It confirmed that VitD supplementation (2000 IU/daily), with or without *n*-3 FAs, decreased hs-CRP by 19% at year 2, although the reduction was attenuated at 4 years. Other inflammatory biomarkers (IL-6, IL-10, and TNF-α) were not significantly altered at year 2 or year 4, while *n*-3 FAs, with or without VitD3, did not significantly affect these biomarkers at either time point. Therefore, the authors partially confirmed a potential role of VitD supplementation in modulating the chronic inflammatory process, systemic inflammation, and possibly autoimmune disease progression [114].

### 4.10. Vitamin D and Dysbiosis

Dysbiosis and gut microbiota dysfunction may result from the interplay of several factors, and chronic inflammation represents a possible drive for them. HIV-1 infection is an example of a chronic disease where VitD seems to counteract dysbiosis. Indeed, patients with HIV-1 infection are characterized by persistent immune activation led by lipopolysaccharides (LPS) and microbial products migrating through a defective gut mucosal barrier. They often present with hypovitaminosis D, and, as a consequence, a concomitant dysregulation of the gut microbiota has been described. The immunomodulatory function of VitD seems to be exerted through the induction of the antimicrobial peptide LL-37, which is protective via the activation of autophagy, the inhibition of HIV-1 transcription, and pathogen killing [115]. Moreover, preclinical and clinical studies demonstrated a potential role of VitD in stabilizing the epithelial tight junction, as well as regulating the intestinal microbiota composition, reducing pro-inflammatory intestinal probacteria. Based on these premises, Missalidis et al. evaluated whether VitD or phenylbutyrate (PBA) may have modulatory action on microbiota composition, metabolite production, and immune activation. As a result, VitD and PBA supplements increase VitD levels, without having a significant improvement in the circulating antimicrobial peptide LL-37 and an actual impact on microbiota [115]. Another chronic inflammatory condition linked to gut microbiome dysbiosis is osteoarthritis (OA). Considering that microbiota dysfunction may lead to chronic low-grade systemic inflammation through LPS, and that LPS is increased in OA patients with low-grade inflammation, Ramasamy et al. conducted a pilot study evaluating the interplay between microbiome, knee OA (KOA), and VitD levels, with the ultimate object of establishing whether VitD deficiency is associated with microbiome dysfunction. In the cohort, specific bacteria were identified in VDD subjects compared to normal controls, and KOA_VDD patients showed specific bacteria species that were not identified in KOA without VDD, suggesting a possible influence of VitD on gut microbiome, depending on the KOA status, and a microbiome influence of the KOA condition in VDD subjects. However, in the KOA_VDD group, the core bacteria from KOA or VDD were not identified, suggesting a possible interplay of both conditions in microbiota dysbiosis [116]. Keeping in line with the potential role of VitD supplementation on microbiota composition in patients with different chronic conditions, Kanhere M et al. demonstrated a different gut microbiota composition in patients with VDD and cystic fibrosis compared to patients with normal VD levels; more specifically, potentially pathogenic species were identified in the microbiome of VDD subjects. Moreover, favorable changes in bacteria species were seen in the VDD group after supplementation compared to the placebo group [117]. The effect of VitD supplementation on gut microbiota in healthy individuals has also been studied. In their RCT, Singh et al. showed a significant increment of health-promoting gut probiotics after VitD supplementation [118]. This finding supports the potentially beneficial role of VitD supplementation in microbiota health. Considering how much VitD is related to several chronic inflammatory diseases and how negatively low-grade chronic inflammation impacts general health, adequate VitD storage should be a priority.

**Table 1:** Clinical studies reporting the effects of Vitamin D supplementation on hallmarks of aging.

Hallmark	Study	Cohort	Aim	Conclusion	Refs.
Genomic instability	Placebo-controlled	92 patients with colorectal adenoma	Evaluate the effect of VitD supplement with 800 UI/day for 6 months	Significant increase in Bax expression (apoptosis promoter); no increase of Bcl-2 expression (apoptosis inhibitor) in VitD group	[61]
	Randomized Clinical Trial	92 subjects with VitD insufficiency, with and without T2DM	Evaluate the effect of VitD supplement with 2000 UI/day for 3 months	Decreased percentage of DNA damage and oxidative parameters when compared to the control group	[62]
Telomere Attrition	Twins UK Cohort ObservationalRegistry	2160 women (aged 18–79) from population-based cohort of twins	Evaluate the relationship between VitD concentrations and the rate of telomere attrition in leukocytes	Positive correlation between serum 25(OH)D levels and telomere length	[64]
	Nurses’ Health Study Observational Registry	1424 women	Evaluate the association between both 25(OH)D and 1,25(OH)2D and leukocyte telomere length	Positive correlation between serum 25(OH)D concentrations and leukocyte telomere length	[65]
	Cross sectional analysis from the Health Professional Follow-Up study	2843 men (from studies of telomeres and cancer)	Evaluate the association between 25(OH)D, 1,25-dihydroxyvitamin D (1,25(OH)2D) and leukocyte telomere length	No association between VitD levels and leukocyte telomere length	[66]
	Cross sectional analysis from Northern Finland Birth Cohort 1966	5096 younger adults	Evaluate associations between 25(OH)D and body mass index (BMI) with leukocyte telomere length and whether associations are independent of concentrations of C-reactive protein	No association between VitD levels and leukocyte telomere length	[67]
	Cohort from the Newcastle 85+ Study	775 older adults (>85 ys), community dwelling, and institutionalized older patients	Evaluate the association between serum VitD concentration and telomere length in blood cells at baseline, 18 and 36 months	Significant positive association between VitD and telomere length at baseline, but inconsistent relationships at subsequent time points	[68]
	Case–control study	African American Gullah women with systemic lupus erythematosus (*n* = 59) and healthy controls (*n* = 59)	Evaluate the relationships between VitD status, telomere length, and anti-telomere antibodies	Significant association between shorter telomeres and lower 25(OH)D levels in patients and healthy control; shorter telomeres at follow-up in VitD-deficient patients	[69]
	Retrospective case–control study	62 stable hemodialysis patients and 60 controls	Evaluate the potential protective role of VitD supplementation on telomere length in peripheral mononuclear cells	Hemodialysis patients treated with VitD had greater telomere length in peripheral mononuclear cells	[70]
	Double-blind, placebo-controlled clinical trial	37 overweight African American subjects (18 placebo and 19 intervention)	Evaluate the effect of VitD3 oral supplementation (60,000 IU/month)	VitD supplementation significantly increased telomerase activity	[74]
Epigenetic alteration	Sub analysis from RCT	51overweight/obese African Americans VitD-deficient subjects	Evaluate different effects of VitD3 supplement (600 IU/day, 2000 IU/day, and 4000 IU/day) for 16 weeks compared to placebo	DNAm age was correlated with chronological age in patients with low VitD levels, while the supplementation was able to decrease DNA metilation and slowed epigenetic aging	[75]
	Southampton Women’s Survey: subgroup analysis from prospective cohort	230 children from the Southampton Women’s Survey (SWS) mother–offspring study	Evaluate the correlation between VitD status and Retinoid-X receptor-alpha (RXRA) methylation in umbilical cord DNA	Methylation at RXRA locus in the umbilical cord was associated with the offspring bone mass; specific methylation at one locus was associated with maternal VitD deficiency	[78]
	The Maternal VitD Osteoporosis Study (MAVIDOS RCT): subgroup analysis	479 received VitD3 supplementation and 486 were in the placebo group	Evaluate DNAm at a specific site in the RXRA locus (10 CpG)	In the cohort of mothers’ receiving VitD, the methylation levels in the RXRA-specific site were significantly lower compared to the placebo group	[79]
	Randomized controlled pilot study	Pregnant women receiving VitD3 400 IU (*n* = 6, control group) or 3800 IU (*n* = 7, intervention group)	Identify differentially methylated loci among infants born to mothers supplemented with VitD 400 IU/day (i.e., control) versus 3800 IU/day (i.e., intervention)	Compared to control, intervention was associated with mean 25(OH)D levels reflective of sufficient maternal VitD status by birth, as well as significant gains in maternal leukocyte DNA methylation associated with genes involved in cell migration/motility, development, and growth	[80]
Cellular senescence	Case–control study	120 individuals (30 young, 30 older, and 30 older in treatment group 1 (1 sachet of Difensil^®^ IMMUNO/day for 12 weeks) and 20 older in treatment group 2 (2 sachet of Difensil^®^ IMMUNO/day for 6 weeks)	Evaluate the effect of the nutraceutical supplement (comprehensive of Sambucus nigra, zinc, tyndallized Lactobacillus acidophilus (HA122), arabinogalactans, VitD, vitamin E, vitamin C, and group B vitamins) on specific signatures of immunosenescence	Older people treated for 30 days improved IL-6, CRP, and lymphocytes levels independent from the dosage of the supplements used; despite the improvement, they were not able to reach the same conditions of young patients	[95]
	Double Blind RCT	110 older fit or frail subjects	Evaluate the effect of alphacalcidol supplementation in inflammatory profile (Il-6, IL-10, TNF, CD4/CD8 ratio, CD8+, and CD28-)	Cohort receiving alphacalcidiol had a better immune profile across the whole cohort as well as the cohort divided according to frailty status	[96]
Chronic inflammation	VITAL study sub-cohort	25,871 women aged ≥55 and men aged ≥50	Measure the effect of VitD supplementation (2000 UI/day) and/or *n*-3 FAs on systemic inflammatory biomarkers	VitD supplementation with or without *n*-3 FAs decreased hs-CRP by 19% at year 2, and the reduction was attenuated at 4 years Other inflammatory biomarkers (IL-6, IL-10, and TNF-α) were not significantly modified	[114]
Dysbiosis	Sub analysis from a RCT	167 HIV-1 patients receiving VitD 5000 IU/day and 500 mg phenylbutyrate (PBA) for 16 weeks	VitD or PBA may have a modulatory action on microbiota composition, metabolites production, and immune activation	No significant improvement in circulating antimicrobial peptide LL-37 and an actual impact on microbiota	[115]
	Pilot study	24 patients with knee osteoarthritis (KOA), healthy controls, with and without VitD deficiency	Evaluate the interplay between microbiome, knee OA (KOA), and VitD levels for establishing whether VitD deficiency is associated with microbiome dysfunction	Patients with KOA and VitD-deficient showed specific bacteria species, absent in KOA, and not VitD-deficient; The different core bacteria in KOA and VitD-deficient suggested an interplay between conditions	[116]
	RCT	41 patients with cystic fibrosis divided in VitD insufficient (*n* = 23) and VitD sufficient (*n* = 18)	Comparing microbiota composition in VitD insufficient and sufficient patients and evaluate the impact of VitD supplement.	Potentially pathogenic species were identified in the microbiome of VitD-deficient subjects	[117]
	Interventional study	100 healthy women	Evaluate gut microbiota composition before and after VitD supplementation	Significant increment of health-promoting gut probiotics after VitD supplementation	[118]

## 5. Discussion and Future Perspectives

Aging is a complex and multifactorial process, marked by the progressive decline in organ functions, leading to frailty and increased susceptibility to age-related diseases. Although years of research have supported the importance of each hallmark in the process of aging, a significant amount of recent evidence indicates that the hallmarks are not acting in isolation but rather have complex, interconnected interactions. Hallmarks of aging are a set of interconnected biological mechanisms that, individually or synergistically, contribute to molecular and cellular damage, potentially initiating and accelerating the onset of age-related diseases. The interconnection among hallmarks of aging highlights the need to consider aging holistically and investigate biological and phenotypical changes integrally, ultimately disentangling the potential agonistic, antagonistic, and modulator factors leading to organ disease. Currently, we are not certain what impact hallmark interactions have on normal or pathological aging or if there are possible hierarchies among the hallmarks. In addition, whether there is a threshold beyond which such interactions could overwhelm compensatory mechanisms needs to be investigated [15]. Given the epidemiological scenarios and the available scientific and technological achievements, it seems pivotal to move and integrate the routes of research from the biological to the clinical layers in the continuum of dynamism from integrity to damage, from vitality to frailty, with the ultimate goal of expanding a valuable lifespan. In this perspective, it appears a priority to explore the efficacy of available multi-target interventions with potential for modulating the hallmarks of aging, then verifying the impact on the overall body’s homeostasis and functioning. Indeed, the hallmarks of aging could represent a milestone to be addressed and understood in healthy aging studies and interventions, hopefully driving the transition from preclinical contexts to clinical settings.

In light of promoting active longevity, strong evidence supports the deleterious effects of hypoVitD on musculoskeletal health, particularly in causing falls, fragility fractures, and mobility issues. Beyond its musculoskeletal effects, VitD may exert several potential extra-skeletal actions, modulating several biomolecular mechanisms and pathways that have been identified as VDR-related in a wide range of cellular types and at multiple cellular layers. Among the biomolecular mechanisms, the hallmarks of aging are pivotal to disentangle the modulatory or causal effects of VitD on the onset and progression of aging and age-related diseases. VitD appears to regulate and influence several hallmarks of aging through genomic and non-genomic pathways, influencing their expression directly or indirectly by activating epigenetic changes or just influencing the cellular environmental milieu. Based on the available clinical studies, we can conclude that ViD may contribute to genomic stability, but it does not seem to have an impact on telomere length, although some findings support a protective role of VitD on telomerase activity in obese individuals. Encouraging evidence supports the epigenetic effects of VitD, which may be able to control and attenuate DNA damage in adults, infants, and their mothers. Then, some evidence supports the protective role of VitD in modulating senescence and related-SASP, but proves concerning the capacity of VitD to lower chronic inflammation in the long term are still waiting to come, as well as those showing better intercellular communication. Calcium is also recognized as a potential regulator of senescence in vivo and plays a key role in several processes in which senescence is involved, such as wound healing, cell migration, and invasion, as well as insulin sensitivity [119], suggesting that calcium and senescence may be functionally linked in many contexts [120]. To date, there is no evidence for an impact of VitD on attenuating SC exhaustion, but it looks promising that VitD modulates the vitality and efficacy of SC infusion. However, caution is needed in interpreting these findings, considering the novelty and the debate about this approach. Notably, clinical studies support the potential of VitD supplementation in hampering dysbiosys, prompting the recovery of gut microbiota health, and providing multiple health benefits.

Despite the interest in VitD supplementation as a strategy supporting human longevity and some evidence about its potential in modulating hallmarks of aging, we are still far from the point of translation from bench to bed. With some exceptions, the majority of human studies investigated the impact of VitD supplementation on the single hallmark of aging by using disease models or peculiar subgroups of the population, focusing mainly on inflammatory-related changes without exploring the correlations with other hallmarks or functional or biological markers of aging. Therefore, current knowledge does not allow yet to define a potential optimal VitD dosage for modulating hallmarks of aging or the chronological or functional age at which VitD supplementation might be useful in modulating aging biologically related factors. However, aging is a progressive process that starts soon after achieving the structural and functional body’s maturation. It mainly consists of adaptative changes that lead over time to the accumulation of deficits and insults. Then, we could speculate that early interventions to avoid VitD insufficiency might be protective in the long run, but longitudinal studies based on homogenous cohorts and targeted therapy are warranted.

Indeed, there are few intervention studies conducted in humans that confirm potential pathways already explored in preclinical models. Human studies carried out so far are mainly transverse, and those few longitudinal studies were too small, limited by a short follow-up time, or focused on a single biomolecular mechanism, unable to obtain clarifying results or provide a wide perspective on the overall cellular and pathophysiological changes. Moreover, many literature discrepancies exist and may be justified by the use of different VitD supplements, different dosages, heterogeneous VitD baseline levels, often even unknown, different measurement techniques for hallmarks, and, ultimately, different target populations and aims.

From a clinical standpoint, it is a relevant approach to preventing VitD deficiency at any age since it is associated with osteomalacia and ensuring a sufficient level of VitD in the general population. It is strongly recommended that seniors with a fall history or those living in long-term care institutions maintain or regain optimal VitD levels by means of supplementation (at least 800–1000 IU daily). However, optimal VitD levels are recommended in adults or older adults at high risk for fragility fractures. Supplementation with VitD is a first-line intervention in the secondary prevention of falls and fragility fractures, and, in these cases, it is advisable to measure the patient’s serum VitD concentrations after two-to-three months of ViD supplementation. Indeed, there is no direct evidence supporting a clear advantage in performing a basal assessment of VitD at the levels reached with supplementation. On the contrary, the failure or inadequate supplementation with vitamin D in patients on antiresorptive or anabolic treatment for osteoporosis is thought to further amplify the problem and expose patients to a high risk of re-fracture and mortality [121].

In summary, a growing body of research has explored the relationship between VitD and some hallmarks of aging, with varying results depending on study subgroups and design. Despite encouraging routes for potential interventions based on VitD, further investigations are required to elucidate the effects from an integrated perspective and set the pillars for the potential clinical implications of these findings.
